# Effect of primary Phacoviscocanalostomy/ Viscocanalostomy on intraocular pressure of normal tension glaucoma patients: 3-year results

**DOI:** 10.1186/s12886-017-0596-y

**Published:** 2017-11-06

**Authors:** Derek Kwun-hong Ho, Adesuwa Garrick, Seemeen Aazem, Divya Mathews

**Affiliations:** Stanley Eye Unit, Abergele Hospital, Llanfair Road, Abergele, Conwy, LL22 8DP UK

**Keywords:** Non-penetrating, Viscocanalostomy, Phacoviscocanalostomy, Normal tension glaucoma, Trabeculectomy

## Abstract

**Background:**

The objective of this study was to evaluate the efficacy of Viscocanalostomy/Phacoviscocanalostomy (VC/PVC) in lowering intraocular pressure (IOP) in Normal Tension Glaucoma (NTG) patients.

**Methods:**

Retrospective electronic database review of patients who underwent VC/PVC for NTG between December 2009 and November 2011 at Stanley eye unit in Abergele Hospital. Goldmann applanation tonometry (GAT) was used for all IOP measurements which were taken at the time of listing for surgery and at 1 day, 1 week, 1 month, then 3-monthly up to 1 year, then half-yearly up to 3 years post operatively. Statistical analysis was performed using unpaired t-test. A *P* value of <0.05 was accepted as the level of significance.

**Results:**

Operations were performed on 94 eyes from 67 patients over the study period. The mean age at the time of surgery was 76.4 years. Pre-operative IOP was 17.75 ± 2.19 mmHg (range 12–21 mmHg). 3 year follow-up showed a mean IOP of 13.41 ± 2.22 mmHg (range 8–18 mmHg). By year 3, 17 patients needed laser goniopuncture and 25 patients needed antiglaucoma drops. IOP was reduced by 24.4% at 3 years post-surgery, which was statistically significant (*p* < 0.0001).

**Conclusions:**

From our results, which show a 24.5% reduction in IOP at 3 years with 12% complication rate, we propose that a logical surgical management for NTG patients would be viscocanalostomy, thereby keeping trabeculectomy as an alternative.

## Background

Glaucoma is a progressive optic neuropathy which may be due, in part, to an elevated intraocular pressure (IOP) with pathognomonic changes in the optic disc and visual fields.

Normal tension glaucoma (NTG), a type of primary open angle glaucoma, can be defined with the following criteria [1].

1. A mean IOP off treatment consistently equal to or less than 21 mmHg on diurnal testing, with no single measurement greater than 24 mmHg.
2. Open drainage angles on gonioscopy
3. Absence of any secondary cause for a glaucomatous optic neuropathy
4. Typical optic disc damage with glaucomatous cupping and loss of neuroretinal rim
5. Visual field defect compatible with the glaucomatous cupping (disc/field correlation)
6. Progression of glaucomatous damage.

Apart from a low/normal IOP, NTG patients are more likely to have systemic microvascular or circulatory abnormalities, such as migraine, exaggerated vasospastic response to cold recovery test, higher incidence of cerebral vascular ischemia, systemic hypertension or hypotension, or other vascular abnormalities like disc haemorrhage and peripapillary atrophy [2].

Following a diagnosis of NTG, (as in all forms of glaucoma) the first line of treatment is antiglaucoma drops to reduce the IOP to low teens or, in some cases, single digits. Progression, despite treatment, warrants additional antiglaucoma drops. Surgical intervention is the next option should topical treatment fail to slow progression. In the past, trabeculectomy was the only surgical option for progressive glaucoma. More recently, the concept of ‘non-penetrating’ glaucoma surgery has gained interest for its potential to limit some of the complications associated with more invasive procedures to lower IOP [3]. In the mid 1990’s, Stegmann et al. introduced viscocanalostomy (VC) and reported successful results in glaucoma patients from Africa [4].

The advantage of doing VC is that it can also be combined with cataract surgery (PVC) with good surgical outcomes. In comparison, phacotrabeculectomy has more complications and requires more intervention / manipulation to ensure success [5].

In VC, entry into the anterior chamber is avoided. This prevents over filtration, hypotony and as a result fewer post-operative follow up visits. However, there have been debates on the longevity as well as the efficacy of VC [6].

Few publications reported on the efficacy of VC/PVC in NTG patients. The objective of this study was to evaluate the efficacy of VC/PVC in lowering IOP in NTG patients.

## Methods

Patients who underwent VC/PVC as their primary glaucoma surgery for NTG between December 2009 and November 2011, at the Stanley eye unit in Abergele Hospital, were identified from the intranet database retrospectively with no control arm. The study followed the tenets of the Declaration of Helsinki. The indication for surgery was progressive optic neuropathy, or visual fields, in patients on maximum topical antiglaucoma drops or could not tolerate antiglaucoma drops, with IOP below 21 mmHg. All procedures were performed by a single surgeon (DM) under Subtenon’s anaesthesia. VC/ PVC procedures were performed by the same surgeon as previously described [4].

Post-operative follow-up for non-complicated cases was on day 1, week 1, month 1 and then 3-monthly for the first year. In the immediate post-operative period, patients had topical steroids (G. prednisolone acetate 1%) on a reducing regimen and antibiotics (G. chloramphenicol 0.5%) for 4 weeks. IOP was measured with Goldmann Applanation Tonometry (GAT) at every visit and visual field was repeated 3 months after surgery. Laser goniopuncture (LGP) was performed if there was an increase in IOP. If an immediate drop in IOP was not achieved, antiglaucoma drops were commenced.

Statistical analysis was performed using unpaired t-test and a *P* value of <0.05 was accepted as the level of significance.

## Results

Ninety four eyes from 67 patients were identified, 30 of whom were male and 37 were female. The mean participant age was 76.4 ± 9.1 years (range 50–90 years). Twenty five eyes had VC, of which 19 have previously undergone cataract operations, and 69 eyes had PVC. In total, 10 patients (11%) had inadvertent perforation of the Trabeculo-Descemet’s Window (TDW). The average number of topical medication prior to surgery was 2.70 ± 0.91, with 8.9 ± 5.9 years on topical antiglaucoma medication (range from 2 months to 27 years). From the database we also identified additional 4 eyes who had previous trabeculectomy and 1 had vitrectomy/cataract surgery. These were excluded from the study.

**Fig. 1:**
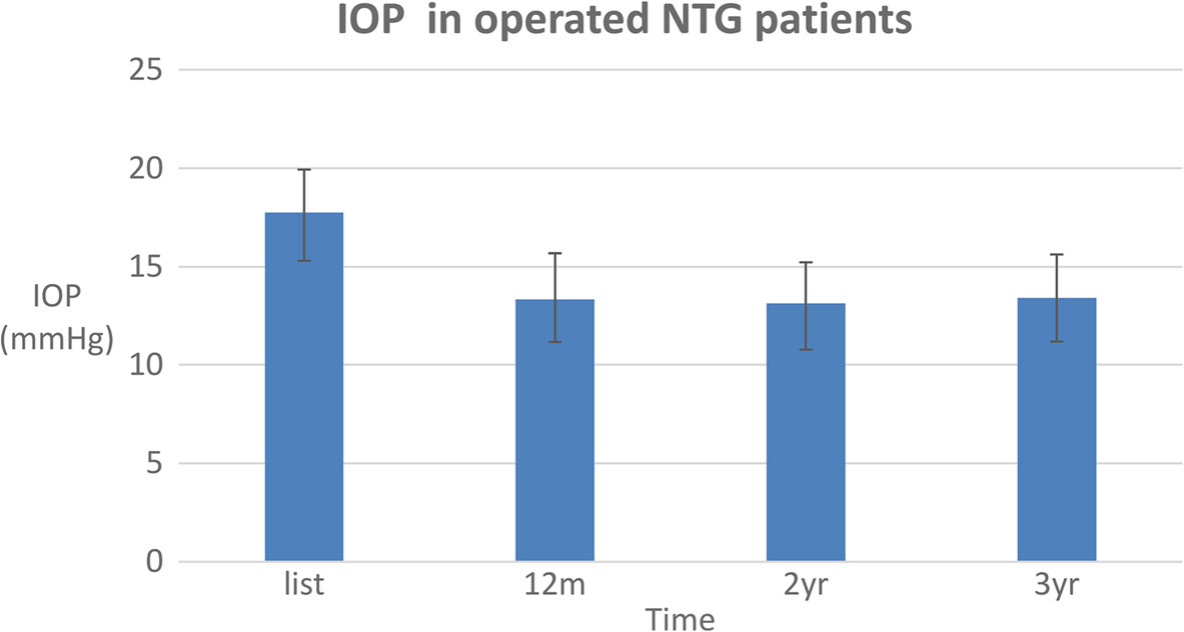
Mean pre- and post-operative IOP following VC/PVC in NTG patients (m = month; yr. = years)

Mean pre-operative IOP was 17.75 ± 2.19 mmHg (range 12–21 mmHg). Post-operative IOP at 6 months was 12.69 ± 2.05 mmHg (range 8–19 mmHg) with one patient needing laser gonio-puncture (LGP). At 12, 24 and 36 months, mean IOP was 13.34 ± 2.35 mmHg (range 8–24 mmHg), 13.13 ± 2.10 mmHg (range 9–18 mmHg) and 13.41 ± 2.22 mmHg (range 8–18 mmHg) respectively (Fig. 1). This represents a 24.5% reduction in IOP over 3 years.

Nine patients needed LGP and 4 patients were prescribed anti-glaucoma drops by year 1 as their IOP was suboptimal. By year 2, 16 patients were prescribed anti-glaucoma drops and 11 patients received LGP treatment. By the end of study period at year 3, 17 patients had LGP and 25 patients were given anti-glaucoma drops (Table 1).

**Table 1:** Pre and post-operative IOP, with percentage reduction, at 12, 24 and 36 months

Time	Number of eyes (loss to follow-up)	Mean IOP (SD) (mmHg)	Percentage IOP reduction (%)	Number of eyes received laser goniopuncture (LGP)	Number of eyes receiving antiglaucoma drops
Pre-op	94 (0)	17.75 (±2.19)	N/A	N/A	N/A
12 months	82 (12)	13.34 (±2.35)	24.8%	9 (10%)	4
24 months	79 (15)	13.13 (±2.10)	26.0%	11 (12%)	16
36 months	75 (19)	13.41 (±2.22)	24.5%	17 (18%)	20

There were 12 patients (13%) lost to follow-up by 12 months. This increased to 15 (16%) by year two and 19 (20%) by the end of the three-year study period. At 12 months, percentage decrease compared to the pre-operative IOP was 24.8%, at 24 months the decrease was 26.0% and at 36 months 24.5%. Statistical analysis using unpaired t-test showed that IOP reduction was significant (*p* < 0.0001) (Table 1).

**Fig. 2:**
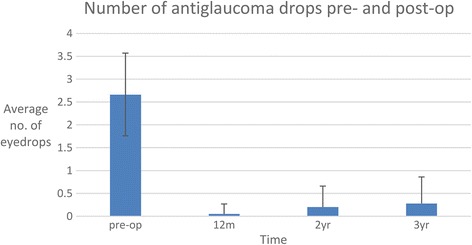
Median number of anti-glaucoma medication prescribed to NTG patients pre- and post-operatively (m = month; yr. = years)

The number of anti-glaucoma drops used at 36 months was 0.28 ± 0.58 compared to 2.66 ± 0.91 pre-operatively, 0.05 ± 0.22 at 12 months and 0.20 ± 0.46 at 24 months (Fig. 2). Fifty eight operated eyes (62%) were drop free at 36 months follow-up.

As at 36 months follow-up, 17 eyes (18%) received LGP for sub-optimal IOP. Median time to perform LGP following VC/PVC was 12 months (range 6–36 months). Mean IOP by year 3 in this sub-group was 13.65 ± 2.23 mmHg, reducing from the listing IOP of 18.88 ± 1.90 mmHg, representing a reduction of 28%. 10 (59%) of these 17 eyes were also commenced on anti-glaucoma drops, achieving an average IOP of 13.30 ± 2.36 mmHg at 3 years. This compares to the 7 (41%) out of the 17 eyes, which maintained an average IOP of 14.14 ± 2.12 mmHg without drops.

10 (11%) of 94 operated eyes had inadvertent intra-operative perforation of TDW. In this group, pre-operative IOP was 18.4 ± 1.8 mmHg, reducing to 12.3 ± 1.7 mmHg at 6 months, 13.7 ± 1.9 mmHg at 12 months, 11.7 ± 2.8 mmHg at 24 months and 12.5 ± 2.7 mmHg at 36 months. Two patients needed LGP at 12 months and 4 were prescribed antiglaucoma drops by 36 months. Analysis using the unpaired t-test showed no statistical significance (*p* = 0.17) in IOP difference at 36 months, in the patients that had inadvertent perforation of the TDW and those that had an intact TDW, with 36 months IOP of 13.5 ± 2.1 mmHg.

**Fig. 3:**
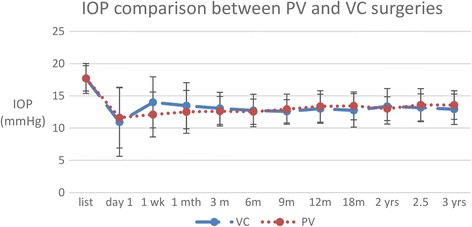
Mean pre- and post-operative IOP following PVC and VC (m = month; yr. = years)

Comparing the pseudophakic patients who underwent VC and the patients who were rendered pseudophakic from their PVC surgeries, listing IOP for PVC patients was 17.70 ± 2.33 mmHg while VC patients IOP was 17.72 ± 1.96 mmHg (*p* = 0.97). By the end of the 3 year study period, final IOP were 13.58 ± 2.22 mmHg and 12.92 ± 2.36 mmHg for PVC and VC patients respectively (*p* = 0.27). This represents a reduction of 4.1 mmHg for PVC and 4.8 mmHg for VC cases (Fig. 3). No significant differences in post-operative IOP between the two groups were demonstrated.

Overall, 1 patient needed conjunctiva resuturing at 1 week due to persistent leak. One patient required anterior chamber washout for hyphema 3 days post surgery and 1 other patient developed post operative cystoid macular oedema at 4 weeks. Four patients developed cystic blebs in spite of tight suturing of the flap possibly due to either thin superficial flaps or early suture dehiscence and scleral wound gape; however none of them required bleb refashioning.

At 36 months, the average Mean deviation of visual field defect was −15.17 ± 15.71 (range − 2.1 to −111.3) compared to an average of −11.77 ± 6.76 (range − 30.18 to −1.88) before surgery. Visual field analysis using unpaired t-test gave a *p*-value of 0.11 (not statistically significant).

## Discussion

The collaborative normal tension Glaucoma study reported slower rate of incident visual field loss in cases of 30% or more reduction in IOP in patients with NTG [7]. This is also supported in a paper by Bhandari et al. (8).

Topical antiglaucoma drops and/laser trabeculoplasty should be used if IOP can be maintained at 30% or more below baseline [8, 9]. In a paper published by the NTG study group titled Intraocular pressure reduction in NTG patients, 57% of the patients achieved a 30% reduction in IOP with topical medication and/with laser trabeculoplasty while the other 43% needed as single fistulising procedure [10]. In contrast, Kamal and Hitchings are of the opinion that fistulising surgery with the use of an antiproliferative agent is the treatment most likely to achieve desired IOP reduction in NTG. They also recognise the risk of post-operative hypotony [1].

One might argue that our study involves a different cohort of patients, without any control group compared to other study populations making it difficult to interpret the results. Bearing this in mind our results show that IOP reduction of 24.4% at 36 months with VC/PVC in NTG patients was comparable to that achieved with trabeculectomy. Hitchings et al. reports a reduction on IOP in NTG patients at just above 30% following trabeculectomy [11]. Similarly, Fontana et al. quotes a 25–30% reduction [12]. Membrey et al., at 2 years review, had a median reduction in IOP of 17 mmHg following trabeculectomy with 5 flourouracil and 15.3 mmHg following trabeculectomy with MMC [13]. The median results in our study was lower at 14 mmHg in year 1 and 3 with 13 mmHg in the 2nd year.

LGP is done if IOP was increasing or if visual field progression was noticed. LGP has the potential to augment aqueous outflow through the TDW [14]. It is preferable to perform LGP rather than commencing topical medication (‘rather too early than too late’) [14]. 17 (18%) of the 94 operated eyes had LGP by 3 years with a mean time to LGP from surgery of 19.9 ± 10.8 months. This is less than the 45% quoted for deep sclerectomy by Ambressin et al. with time to LGP from surgery of 12.4 ± 10 months. Our reduction in IOP post-LGP was also greater, at 28%, than the 20% quoted [15]. Ten patients (59%) were commenced on topical antiglaucoma medication as reduction in IOP following LGP was not sustained.

Fistulising procedures have documented complications. Schultz et al. reported that hypotony occurred in 30% of patients during the post-operative follow-up period [16]. Others reported complications included choroidal effusion, hyphema, hypotony maculopathy, diplopia, bleb leak, blebitis. Song et al. acknowledges the potential complications of trabeculectomy but still maintain that it is the most effective method of achieving low IOPs [3]. One should remember that trabeculectomy also warrants frequent post-operative visits at least weekly in the early post-operative period [17]. And the further use of anti-proliferative agents at least once during the post-operative visit. This translates to a lot of clinic hours spent in the frequent review of these patients in comparison to day 1, 1 week, 4 weeks and 3 months post-operative visits for PVC/VC with no intervention in the form of needling or suture adjustment. Goniopunture may be needed for PVC/VC patients but this is usually one visit any time after 6 months post operatively. The time taken to perform the laser would be similar to do doing a YAG peripheral iridotomy.

Ophthalmic surgeons have often raised concerns on the steep learning curve for PVC/VC. However, once mastered, PVC/VC takes no longer to complete than a trabeculectomy. In our surgeon’s hands, both procedures take the same time. If perforation of the TDW did occur then a surgical Peripheral Iridotomy (PI) was performed as an additional step before completing the rest of the surgical steps of VC. This does not add any significant time to the surgery itself.

Our complication rate was low. Ten eyes (11%) had inadvertent perforation of the TDW. Surgical PI was performed if the iris was presenting. Previous study by Park et al. documented micro-perforation rate of 21.4% and iris prolapsed from ruptured TDW in 5.9% [18]. Similar studies on Non-penetrating glaucoma surgery (NPGS) have reported tearing of the conjunctiva, hyphema, dellen, encapsulated bleb [19]. A recent paper by Grieshaber et al. reports complications following VC including choroidal detachment, gross hyphema and peripheral Descemet’s detachment [20].

We acknowledge that there are a few weaknesses in the study. Firstly it is a retrospective study. In addition the patients in our study are from a different population, without any control group compared to other studies. This might make interpretation of our results with other study populations difficult. Postoperative evaluation including Tonometry was not masked and therefore subject to observer bias. The strengths of this retrospective study includes single surgeon, long term follow-up with a large number of patients compared to similar studies.

Our study did not consider analysis of phakic versus pseudophakic eyes, as our participants included only 6 phakic eyes within the VC subgroup, compared to 88 pseudophakic eyes either from previous cataract operations or as a result of combined PVC surgeries. We considered the number of phakic eyes to be too low to draw any statistical conclusions with sufficient power.

Not analysing visual acuity is one of the weaknesses of this study. However, visual acuity is not a reliable way of judging the success of glaucoma surgery. Also, visual field analysis in previous papers was carried out using various methods, making it difficult to directly compare the results of our visual field analysis.

Membrey et al. observed that the use of MMC in trabeculectomy is associated with a greater risk on visual field progression despite a greater fall in IOP [11]. From our results, a logical surgical management for NTG patients would be viscocanalostomy keeping trabeculectomy as an alternative. We advocate viscocanalostomy for NTG patients where target IOP is in the mid-teens. In our practice, Trabeculectomy has its place in NTG patients where IOP of less than 12 mmHg is desirable. However, we advise cardiovascular workup and ensure compliance in such patients.

## Conclusions

Our study on Viscocanalostomy / Phacoviscocanalostomy operations on 94 eyes with Normal Tension Glaucoma shows 24.4% IOP reduction at 3 years. We propose viscocanalostomy as the surgical management for NTG patients, thereby keeping trabeculectomy as an alternative.

## Abbreviations

GAT: Goldmann applanation tonometry
IOP: Intraocular pressure
LGP: Laser goniopuncture
NPGS: Non-penetrating glaucoma surgery
NTG: Normal tension glaucoma
PI: Peripheral Iridotomy
PVC: Phacoviscocanalostomy
TDW: Trabeculo-Descemet’s Window
VC: Viscocanalostomy

## References

[1] Kamal D, Hitchings R (1998). Normal tension glaucoma--a practical approach. Br J Ophthalmol.

[2] Daugeliene L, Yamamoto T, Kitazawa Y (1998). Effect of trabeculectomy on visual field in progressive normal-tension glaucoma. Jpn J Ophthalmol.

[3] Song BJ, Caprioli J (2014). New directions in the treatment of normal tension glaucoma. Indian J Ophthalmol.

[4] Stegmann R, Pienaar A, Miller D (1999). Viscocanalostomy for open-angle glaucoma in black African patients. J Cataract Refract Surg.

[5] Paul C, Sengupta S, Paul A. Complications of Phacoemulsification vs Phacotrabeculectomy in the treatment of chronic angle closure glaucoma with concomitant cataract. Int J Innov Res Dev. 2013;2(9):58–66.

[6] Hondur A, Onol M, Hasanreisoglu B (2008). Nonpenetrating glaucoma surgery: meta-analysis of recent results. J Glaucoma.

[7] Collaborative Normal-Tension Glaucoma Study Group. The effectiveness of intraocular pressure reduction in the treatment of normal-tension glaucoma. Am J Ophthalmol. 1998;126(4):498–505. 10.1016/s0002-9394(98)00272-4 9780094

[8] Bhandari A, Crabb DP, Poinoosawmy D (1997). Effect of surgery on visual field progression in normal-tension glaucoma. Ophthalmology.

[9] Geijssen HC (1991). Studies on normal pressure glaucoma.

[10] Schulzer M, The Normal Tension Glaucoma Study Group (1992). Intraocular pressure reduction in normal-tension glaucoma patients. Ophthalmology.

[11] Hitchings RA, Wu J, Poinoosawmy D (1995). Surgery for normal tension glaucoma. Br J Ophthalmol.

[12] Fontana L, Viswanathan AC, Poinooswamy D (1997). Surgery for normal tension glaucoma. Target Intraocular Pressure and Visual Field Progression Acta Ophthalmol Scand Suppl.

[13] Membrey WL, Bunce C, Poinoosawmy DP (2001). Glaucoma surgery with or without adjunctive antiproliferatives in normal tension glaucoma: 2 visual field progression. Br J Ophthalmol.

[14] Tam DY, Barnebey HS, Ahmed II (2013). Nd: YAG laser goniopuncture: indications and procedure. J Glaucoma.

[15] Ambresin A, Shaarawy T, Mermoud A (2002). Deep sclerectomy with collagen implant in one eye compared with trabeculectomy in the other eye of the same patient. J Glaucoma.

[16] Schultz SK, Iverson SM, Shi W (2016). Safety and efficacy of achieving single-digit intraocular pressure targets with filtration surgery in eyes with progressive normal-tension glaucoma. J Glaucoma.

[17] Murdoch I (2012). Post-operative management of trabeculectomy in the first three months. Community Eye Health.

[18] Park M, Tanito M, Nishikawa M (2004). Ultrasound biomicroscopy of intrascleral lake after viscocanalostomy and cataract surgery. J Glaucoma.

[19] Suominen S, Harju M, Ihanamäki T (2010). The effect of deep sclerectomy on intraocular pressure of normal-tension glaucoma patients: 1-year results. Acta Ophthalmol.

[20] Grieshaber MC, Peckar C, Pienaar A (2015). Long-term results of up to 12 years of over 700 cases of viscocanalostomy for open-angle glaucoma. Acta Ophthalmol.

